# Elasto-Viscoplastic Material Model of a Directly-Cast Low-Carbon Steel at High Temperatures

**DOI:** 10.3390/ma13102281

**Published:** 2020-05-15

**Authors:** Martin Krobath, Roman Krobath, Christian Bernhard, Werner Ecker

**Affiliations:** 1 Materials Center Leoben Forschung GmbH, Roseggerstrasse 12, 8700 Leoben, Austria; werner.ecker@mcl.at; 2 Montanuniversitaet Leoben, Franz-Josef-Strasse 18, 8700 Leoben, Austria; roman.krobath@unileoben.ac.at (R.K.); christian.bernhard@unileoben.ac.at (C.B.)

**Keywords:** continuous casting, directly-cast microstructure, in-situ bending test, straightening, constitutive model, cyclic viscoplasticity, steel

## Abstract

A model-based process control of material production processes demands realistic material models describing the local evolution of the thermal and mechanical state variables, i.e., temperature, stress, strain, or plastic strain, for the relevant microstructure state. In the present work, a material model for the specific microstructure in a continuously cast strand shell, viable for reproducing cyclic viscoplastic effects, was developed for a 0.17 wt.% C steel. Experimental data was generated using directly-cast samples and a well-controllable testing facility to apply representative loading conditions. Displacement- and force-controlled experiments in the temperature range of 700–1100 °C were conducted, with a special focus on the relevant strain rates documented for the straightening operation. A temperature-dependent constitutive material model combining elastic, plastic, and viscoplastic effects was parameterized to fit the whole set of experimentally-determined material response curves. In order to account for the cyclic plastic material response, a combination of isotropic and kinematic hardening was considered. The material model sets a new standard for the material description of a continuously cast strand shell, and it can be applied in elaborate continuous casting simulations.

## 1. Introduction

The prediction of product quality in steel production is one of the main aims of the ongoing digitalization. This applies in particular to the continuous casting process. The quality of continuously cast steel depends on a wide variety of phenomena, on both a micro- and macroscopic scale. Here, “microscopic” means the order of magnitude of precipitations, dendrites, and grains (nm–µm). These microstructural characteristics depend on cooling conditions and local mechanical deformation and are most relevant for the defect sensitivity of steel at elevated temperatures. In contrast, mass transfer, heat transfer, and deformation in the solid state proceed on the “macroscopic” scale, typically in the length scale of meters. The linking of micro- and macroscopic phenomena in multi-physical models for the casting process is therefore still demanding. The finite element method allows the calculation of the driving forces for the micro phenomena based on the process conditions, see, e.g., Li and Thomas [1] and Liu et al. [2]. The combination of physical models with data-driven approaches is a further step towards overcoming these limitations, see Leitner et al. [3].

Among an unmanageable number of published modelling results for continuous casting, the thermo-mechanical modelling of the behavior of strand shells is one of the most frequently addressed subjects, as the quantification of arising local stresses and strains is a key requirement for defect prediction models. The prerequisite for these thermo-mechanical models is a constitutive material model describing the mechanical material behavior of the steel at temperatures above 800 °C, where steel increasingly exhibits a time-dependent inelastic behavior. Anand [4] proposed a constitutive model, describing rate-dependent deformation of metals at elevated temperatures, but Koric and Thomas [5] showed that the model has limitations in describing the high temperature behavior of steel. Kozlowski et al. [6] introduced constitutive models that relate strain to strain rate, stress, temperature, and steel composition. With respect to these dependencies, they proposed and compared four different elasto-viscoplastic constitutive equations, which are all unable to consider cyclic loading phenomena like kinematic hardening or the Bauschinger effect [7]. Two experimental datasets were used to fit the modelled material behavior: a tensile test data from Wray [8] and a creep test data from Suzuki et al. [9]. Both sources document uniaxial loading cases with reheated specimens, lacking the prevalent microstructure of the continuous casting shell. Despite the deficiencies mentioned, these constitutive models have been used in many finite element models of the continuous casting process since then, see, e.g., Li and Thomas [1], Koric and Thomas [5], and Liu et al. [2]. Zappulla et al. [10] recently simulated solidifying shell mechanics. They explicitly pointed out the lack of material data and mentioned the restrictions of the constitutive models currently applied for strand shell modelling concerning cyclic material effects. Fachinotti and Cardona [11] provide a literature overview on plastic, viscoplastic, and creep constitutive models for continuous casting conditions. The results of the investigated models show a wide dispersion. For comparison with experimental data, they referenced the data of Wray [8] and also highlighted the lack of proper experimental data to describe the high temperature material behavior of the directly-cast material state in a realistic manner to fulfill industrially-demanded accuracy.

Chaboche [12] described a modular set of equations allowing for the constitutive modelling of cyclic time-dependent mechanical material behavior. The model combines viscoplastic flow potentials with isotropic and kinematic hardening evolution laws, considering static recovery terms for kinematic hardening. The constitutive models can be parameterized to cover wide ranges of temperatures and strain rates. The Bauschinger effect can be modelled quantitatively by combining isotropic and kinematic hardening. The model’s complexity arises together with the number of parameters. An accurate description of the mechanical material behavior also demands additional experimental data, especially for considering cyclic material effects. Furthermore, the microstructure of the specimens should be as close as possible to the real strand shell conditions in the continuous casting process. Grain size, grain morphology, inhomogeneities, and precipitations are of highest importance for the deformation behavior of steel at elevated temperatures, as will be shown in detail later on.

This is why in the early 2010s, Montanuniversitaet Leoben started the development of the experimental simulation of surface defect formation under the conditions for the continuous casting of steel. The so-called “In-situ Materials Characterization by Bending” (IMC-B) experiment has the following characteristics:

- Isothermal three-point bending of a sample in-situ, which means directly after solidification and controlled cooling,
- the cooling rate during solidification and thermal cycle is adjusted to the continuous casting process, and
- hence, the microstructure (e.g., grains, grain morphology, and precipitations) corresponds with the strand shell in the continuous casting process.

Krajewski et al. [13] described the first results of this bending test with respect to the formation of surface cracks at temperatures between 700 and 1000 °C, showing significant differences from the results of conventional hot tensile experiments. Minor adjustments to the setup have been established since. Most recently, Krobath et al. [14] published the current IMC-B status, carried out additional tests, and compared the results with respect to the composition of the investigated steel grades.

Besides the results on defect formation, the bending forces measured also depend strongly on the microstructure of the sample. The solidification structure of the IMC-B specimen shows directional dendrite growth, with a coarsening structure with increasing distance from the surface [15]. Therefore, its solidification microstructure is similar to the strand shell, see Presslinger et al. [16]. The microstructural evolution during cooling was investigated by Krobath et al. [17], and they showed the formation of a columnar austenite grain structure. This microstructure again shows similarities to the strand shell, as documented by Reiter et al. [18].

The present paper investigates the mechanical behavior of the solidification microstructure in the relevant temperature and strain rate ranges for a straightening operation of the continuous casting process and provides an elasto-viscoplastic constitutive model, including the full set of material parameters for a representative low-carbon steel.

## 2. Methodology

### 2.1. Experimental Setup

**Figure 1:**
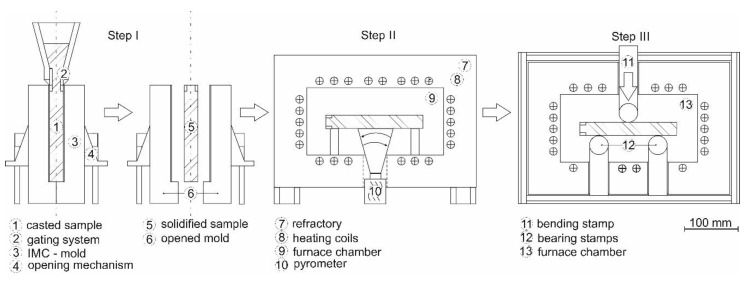
Principle of the “In-situ Materials Characterization by Bending” (IMC-B) test. Step I: melting, casting, solidification, and stripping. Step II: controlled cooling. Step III: isothermal 3-point bending test.

The principle of the IMC-B test is shown in Figure 1. A steel melt with a defined composition is cast into a steel mold that consists of two parts. The controllable heat flux results in a columnar solidification and growth of dendrites and grains; see Krobath et al. [17]. The residual time in the mold for the current testing series is 45 s. Afterwards, the split-mold is opened and the sample is removed with a typical surface temperature of ~1180 °C.

Then, the sample is cooled down to the later bending temperature according to the surface in the casting process. This is realized by a serial connection of chamber furnaces with varying inner temperatures. The surface temperature of the sample is continuously measured with an optical pyrometer. The temperature-time cycle is pre-defined and represents the simulated process. Finally, the sample is placed in bending equipment inside a further furnace and the temperature of the sample is homogenized for 120 s. In the present study, the time between the start of casting and the start of bending amounts to 700 s, which is related to the start of the straightening process of a slab caster with a casting speed of 1.2 m/min and a slab thickness of 225 mm, see Krajewski et al. [13]. The force-displacement curves of the stamp are recorded.

The target composition of the investigated 0.17 wt.% carbon steel is listed in Table 1.

For reasons of comparison, reference bars were cast and treated with the IMC-B equipment, excepting the deformation step. They were cooled down to room temperature with mild cooling conditions of about 1 °C/min, and afterwards they were reheated with a moderate heating rate of about 60 °C/min to the test temperature in a chamber under Ar-flushing to minimize scale formation. The procedure was followed by a temperature homogenization step and a subsequently bending of the “reheated” sample.

Displacement-controlled experiments are conventionally performed to reproduce strand shell deformation during bending and straightening in continuous casting. The stamp displacement and the loading speed applied via cylindrical punch are chosen to resemble the shell deformation parameters during these processes. The bending of the sample leads to a wide spectrum of strain rates of the specimen material, as opposed to uniaxial testing. Whereas this is normally seen as one of the disadvantages of a three-point bending experiment, a model-based evaluation of the experiments provides information about the response of the tested material for a wide range of strain rates. Analytical estimations of strain rates in the straightening stage of the continuous casting process, as performed by Zhang et al. [19], range from around 1 × 10^−4^ to 1 × 10^−5^ s^−1^. In addition to displacement-controlled tests, force-controlled experiments can be conducted. These experiments aim to investigate the creep-dominated material behavior at low external loading and at low strain rates, which is a loading range with a specific lack of experimental data, see Zappulla et al. [10].

**Figure 2:**
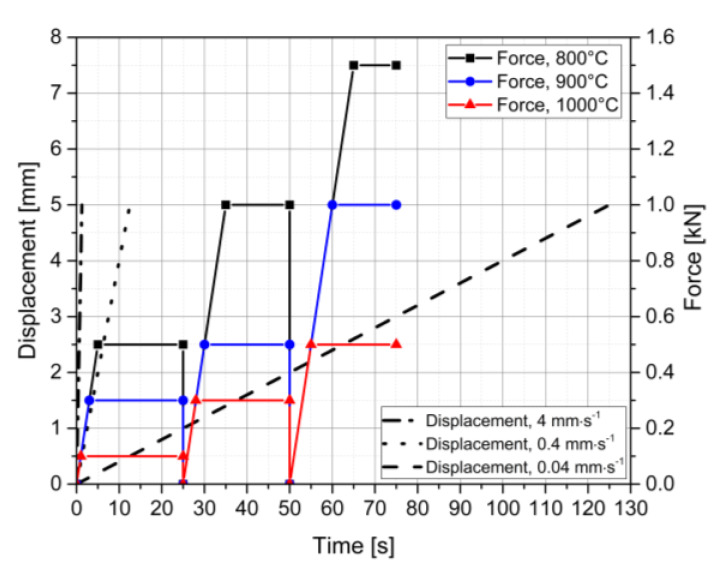
Process control curves for displacement- and force-controlled experiments at 800, 900, and 1000 °C.

The temperatures in the isothermal experiments performed and in the corresponding simulations range from 700 to 1100 °C. These testing temperatures were selected in order to relate to the straightening operation, especially to the conditions on the inner radius surface, which is the position with the highest local loading. Figure 2 shows the loading sequences conducted for the three representative temperatures of 800 °C, 900 °C, and 1000 °C. For the displacement-controlled tests, constant punch velocities of 4 mm∙s^−1^, 0.4 mm∙s^−1^, and 0.04 mm∙s^−1^ are used, resulting in testing times of 1.25 s, 12.5 s, and 125 s, respectively, to reach a displacement of 5 mm. For the test with the slowest velocity, the unloading stage is also recorded for investigation and calibration of the elastic material stiffness. The punch velocity during unloading is again 0.04 mm∙s^−1^. The force-controlled experiments consist of three individual test sequences for each temperature with the same specimen. For the sake of simplicity, these three individual test sequences are displayed as one combined sequence. The virtual connection of the control curves happens at the vertical leaps of the control curves, i.e., at 25 s and 50 s. The force is applied with a constant loading rate of 100 N/s until the defined values are reached. The values depend on the testing temperature, due to the strong dependence of the deformation behavior on temperature. Hence, the different applied forces are dimensioned with respect to the material’s static yield strength at the respective temperatures.

### 2.2. Simulation

All simulations were carried out with the commercial finite element (FE) software package Abaqus [20].

#### 2.2.1. Finite Element Model

**Figure 3:**
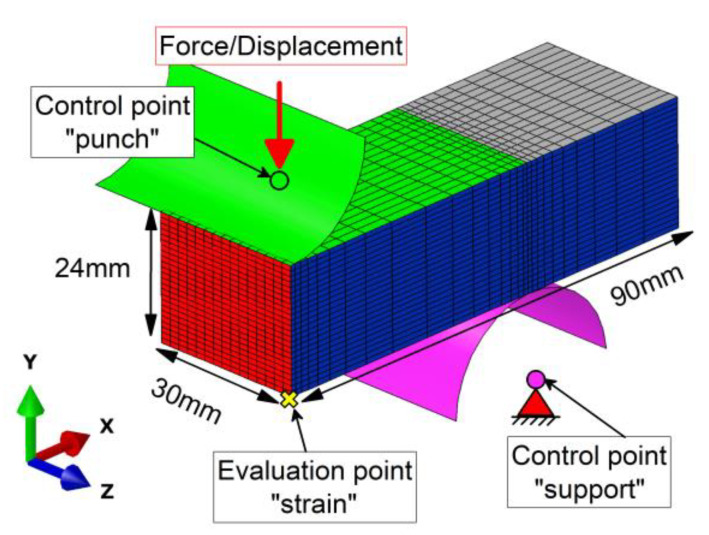
Simulation setup with finite element (FE) mesh, boundary conditions, and loads. The red triangle represents fixed translational and rotational degrees of freedom for the control point “support.” The red vertical arrow represents the applied force or displacement in the y-direction on the control point “punch.” The other translational and rotational degrees of freedom are fixed for the punch as well. The visual surfaces the in x- and z-directions represent symmetry planes to keep computational costs low.

Approximately 10,000 3D hexahedron elements with quadratic shape functions discretize the specimen. The geometric description of the experiment makes use of the sample’s symmetry. All tool components, namely the support and the punch, are modelled as rigid bodies and controlled via one representative control point each. A schematic of the model is depicted in Figure 3. Translational and rotational degrees of freedom of the support are fixed in all directions and for the punch, they are also fixed, except for translation in the y-direction. In the y-direction, a time evolution resembling the experimentally applied displacement or force is prescribed. The mechanical response of the whole sample, and implicitly, the mechanical material response, are also extracted at the control point “punch.” This corresponds to displacement in force-controlled tests and to reaction force, multiplied by four due to the applied symmetry conditions, in displacement-controlled tests. Strain values are analyzed at the evaluation point ”strain” defined in Figure 3, i.e., the central point on the opposite specimen side of the punch contact.

The interaction between the individual tools and the specimen is modelled with surface-to-surface penalty contact and an isotropic coulomb friction coefficient *µ* of 0.2. The mesh density is refined towards the contact regions, see Figure 3. The viscous simulation procedure allows for the modelling of the time-dependent effects of the material model without considering the accelerations as degrees of freedom.

#### 2.2.2. Material Model

The applied material model combines elastic, plastic, and viscoplastic effects and allows the back stresses to recover over time. It contains four modules of Chaboche’s model definition: the time-independent kinematic and isotropic hardening modules in combination with the time-dependent viscoplastic flow and static recovery modules, see [12]. The notation of the material model parameters is adopted from this work, with tensor quantities marked with an underscore. The model’s viscoplastic governing equations are summarized below, in Equations (1)–(6).

Flow function

(1) $$F=\|\underline{\sigma }-\underline{X}{\|}_{H}-R\left(p\right)$$

Viscoplastic potential

(2) $$\Omega =\frac{K}{n+1}\cdot {\left(\frac{F}{K}\right)}^{n+1}$$

Viscoplastic strain rate

(3) $${\underline{\dot{\varepsilon }}}_{p}=\frac{\partial \Omega }{\partial \underline{\sigma }}=\frac{3}{2}\cdot \dot{p}\cdot \frac{{\underline{\sigma }}^{’}-{\underline{X}}^{’}}{\|\underline{\sigma }-\underline{X}{\|}_{H}}\;,\;\;\;\;\;\;\dot{p}={\left\langle \frac{F}{K}\right\rangle }^{n}$$

Kinematic hardening

(4) $$\underline{X}=\overset{3}{\underset{i=1}{\sum }}{\underline{X}}_{i}$$

(5) $${\underline{\dot{X}}}_{i}=\frac{2}{3}\cdot C_{i}\cdot {\underline{\dot{\varepsilon }}}_{p}-\gamma_{i}\cdot {\underline{X}}_{i}\cdot \dot{p}-C_{i}\cdot {\left(\frac{\|\underline{X}{\|}_{H}}{M_{i}}\right)}^{m_{i}-1}\cdot {\underline{X}}_{i}$$

Isotropic hardening

(6) $$R\left(p\right)=R_{0}+Q\cdot \left(1-e^{-b\cdot p}\right)$$

The governing equations depend on the following internal state variables: the accumulated equivalent viscoplastic strain *p*, the viscoplastic strain tensor ${\underline{\dot{\varepsilon }}}_{p}$, and the backstress tensor ${\underline{X}}_{i}$. The parameters *C_i_* and *γ_i_* specify the kinematic hardening characteristics, together with parameters *M_i_* and *m_i_* defining the static recovery effects of the individual backstress tensor ${\underline{X}}_{i}$. The isotropic hardening behavior is characterized by parameters *Q* and *b* and the equivalent viscoplastic strain rate by parameters *K* and *n*.

The material parameters are determined by fitting the force or displacement response for displacement- or force-controlled cases, respectively, of the simulated specimen to the experimentally observed behavior for nine distinct temperature levels. Temperatures range from 700–1100 °C and the loading types correspond to the loading of the experiments, see Section 2.1. The interpolation of parameters between two temperature states is done linearly and no extrapolation strategy is defined. The resulting strain rates are analyzed in detail once the material model parameters are determined. The punch velocity is varied from 4 to 0.4 and 0.04 mm∙s^−1^ to consider the strain rate sensitivity of the material in the displacement-controlled tests. The material model’s response outside the range of strain rates defined by the combination of both loading types is not validated.

#### 2.2.3. Determination of Material Parameters

The material model parameters are determined iteratively, based on user expertise and without the use of optimization routines. The parameters are strongly interdependent and previously applied optimization routines performed poorly in parameter adjustment tests as opposed to manually adjusted parameter sets.

The parameters are determined using a step-by-step procedure for each temperature state. In a first step, the elastic material response is parameterized for both loading types by fitting the early loading and the unloading response where hardly any inelastic effects occur. For force-controlled loading, the individual relaxation steps are analyzed in terms of remaining deformation of the specimen to isolate the reversible elastic material response. The second step is the determination of approximate kinematic (*C_i_* and *γ_i_*) and isotropic (*Q* and *b*) hardening parameters with a focus on the displacement-controlled tests and moderate punch velocities. The mechanical response of the unloading stage reveals a kink, indicating the occurrence of cyclic viscoplastic effects. Reproduction of this behavior is achieved by defined scaling of isotropic and kinematic hardening parameters (Q, b, *C_i_* and *γ_i_*). Step three is the implementation of the dynamic modules (*M_i_*, *m_i_*, *K*, and *n*) with a focus on force-controlled tests for evolution over time and on the deviation between different punch velocities in displacement-controlled tests. The final step is fine-tuning of all parameters using all relevant material response curves and weighting the errors as desired.

## 3. Results

### 3.1. Experiment

**Figure 4:**
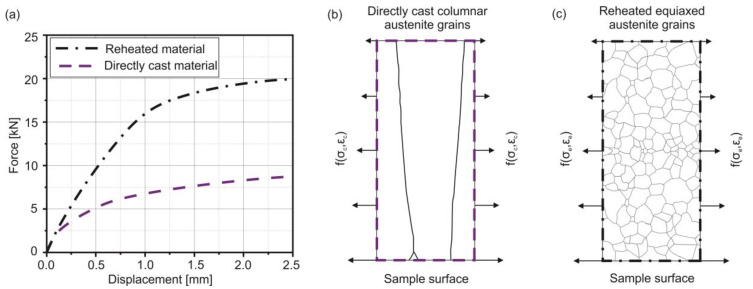
(**a**) Mechanical response of samples from low-carbon steel directly-cast and reheated, (**b**) Schematic view of a directly-cast coarse columnar austenite grain structure [21], (**c**) Schematic illustration of austenitic microstructure of a reheated sample [22].

Figure 4 illustrates the characteristics of the bending response of a directly-cast columnar austenite grain structure in comparison to a reheated microstructure. According to the displacement and the recorded stamp force, Figure 4a shows the different mechanical response of directly-cast and reheated specimens of a low-carbon steel at the same temperature in the fully austenitic range in the IMC-B testing setup. Figure 4b and c visualize the loading direction and schematically, the obvious differences in the microstructure. The recrystallized grain structure is formed during reheating from room temperature to the test temperature and consists of small equiaxed austenite grains, while the casted sample shows coarse columnar grains. Since the force response of the reheated sample is already twice as high as for the directly-cast sample even at small displacements of 1 mm, the development of a viable material model for the actual microstructure of directly-cast material is crucial for accurate mechanical calculations of solid regions in continuous casting strands.

**Figure 5:**
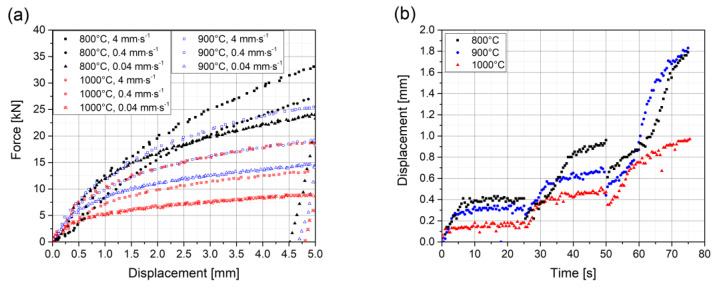
(**a**) Experimental results of displacement-controlled tests. All three strain rates for the three representative temperatures—800, 900, and 1000 °C—are displayed. For the experiments with the lowest punch velocity, the unloading state is also recorded. (**b**) Experimental results of force-controlled tests for the same three temperature states. Note the different force plateaus applied and the well comparable response for the different temperatures.

The results of the experiments are visualized in Figure 5 for three representative temperature states: 800, 900, and 1000 °C. The investigated material shows a significant strain rate sensitivity in displacement-controlled experiments. For the experiments with the lowest punch velocities of 0.04 mm∙s^−1^, the unloading state is also recorded. Detailed analyses of the unloading force displacement curves of the displacement-controlled experiments reveal a slight kink during unloading, indicating the occurrence of a Bauschinger effect.

Note that the experiments with faster punch velocities of 0.4 and 4 mm∙s^−1^ at a temperature of 800 °C show a deviation of the curve’s shape with respect to the other curves, especially at low displacement values. This effect occurs because slight tilting of the specimen cannot always be prevented in experiments, particularly at lower temperatures and higher punch velocities. Hence, the experiments with the aforementioned curve’s shapes are not taken into account for modelling of the evolution of strain hardening. Nevertheless, the strain rate sensitivity of the material in displacement-controlled experiments at 800 °C can be roughly estimated with the prevalent data.

The results of the force-controlled experiments show obvious creep response for the force plateaus. A combination of static and dynamic effects can be noticed for the individual load ramps, especially for the application of the highest load from a time of 50 to 70 s.

### 3.2. Model-Based Parameter Determination

A full set of isothermal constitutive material model parameters is determined for nine temperature grid points from 700 °C to 1100 °C in 50 °C intervals.

#### 3.2.1. Elastic Properties 

**Figure 6:**
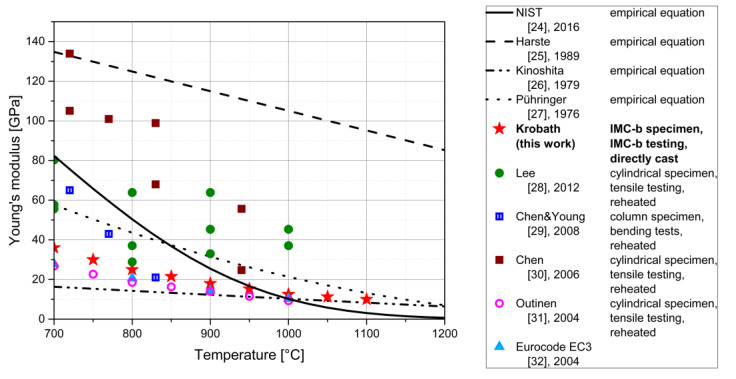
Compilation of a variety of documented Young’s moduli of structural steel at elevated temperatures from 700 to 1200 °C [24,25,26,27,28,29,30,31,32]. The dots represent experimental measurements, whereas the lines represent empirical equations. For experimental measurements, the specimen type, testing type, and microstructural history of the samples are described in the legend box.

The parameterized elastic material response, in terms of Young’s modulus over temperature, is summarized and compared with numerous published values from the literature for structural steels in Figure 6. All of the steels considered in this comparison are low-carbon steels with a carbon content composition between 0.08 and 0.23 wt.% C. The effect of alloying on the Young’s modulus should not be bigger than about 10% [23].

The documented elastic properties in the literature show strong variations in values, as seen in Figure 6, even for the same specimen type, testing method, and microstructural history. The possible reasons for the discrepancies are discussed in the literature. In NIST Technical Note—1907 [24], difficulties in accurate measuring at high temperatures are discussed. The testing facilities are often designed for tests at lower temperatures, i.e., higher loads and, consequently, the strain gauges lack sensitivity in the very low force region for elastic analyses. They propose an uncertainty function which rises linearly with temperature. Another source of measurement or interpretation errors is the viscoplastic behavior of steels at the investigated temperatures. For this reason, a model for the viscoplastic deformation is needed to separate the elastic and viscoplastic displacement contributions and finally determine the Young’s modulus, as demonstrated in the current paper.

In the present case, the testing setup is designed especially for a high temperature regime. The red stars represent the values parameterized in this work. The directly-cast characteristics of the specimen of this work are unique in the range of documented investigations. The values rank closely to the lower limits of the documented values. Still, since the microstructure of the material is significantly different, the values from this work stand for themselves.

The Poisson’s ratios are not determined within this work, as the testing setup makes it hard to isolate transversal contraction effects. The values are calculated using a temperature-dependent empirical equation, as suggested by Kinoshita [26].

#### 3.2.2. Viscoplastic Properties

**Figure 7:**
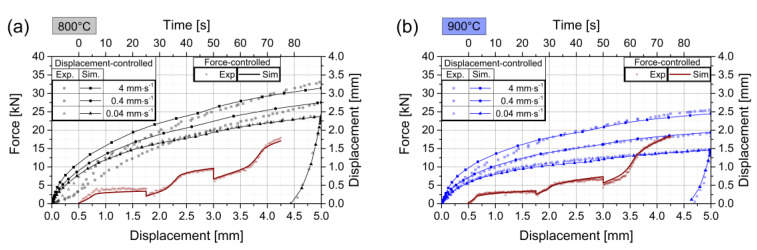

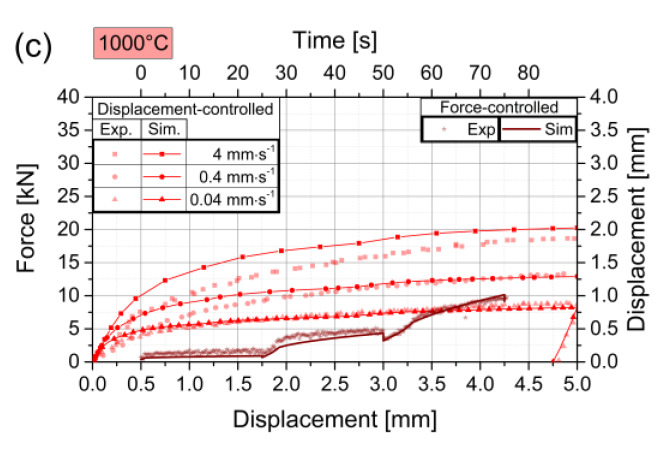
Simulated material response for displacement- and force-controlled conditions in comparison with experimental data. Displacement-controlled curves correspond to the left and lower axes, whereas force-controlled curves correspond to the right and upper axes. The three graphs show the three representative temperatures (**a**) 800 °C, (**b**) 900 °C, and (**c**) 1000 °C.

Figure 7 compares experimental and simulated test results for the three representative temperatures 800, 900, and 1000 °C. The material model describes the material response for all investigated temperature levels well. A small deviation can be recognized in the displacement-controlled tests at 800 °C at low displacements for punch velocities 4 and 0.4 mm∙s^−1^. As mentioned in Section 3.1, the experimental data for this region is not always free from measurement artifacts, especially at lower temperatures. The punch velocity for displacement-controlled tests at 1000 °C shows a small offset between simulated and experimental curves for punch velocity 4 mm∙s^−1^, while all other curves match well.

**Figure 8:**
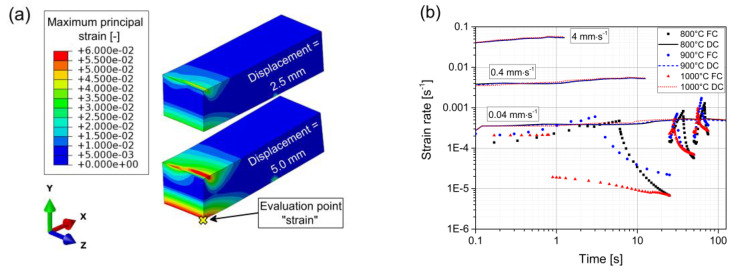
(**a**) Distribution of maximum principal strain in the specimen at 900 °C, punch velocity 0.04 mm∙s^−1^, and two representative displacement states. The evaluation point for strain rate estimations is indicated by a yellow cross. (**b**) Calculated strain rates for all simulations. The loading type abbreviations DC and FC stand for displacement- and force-controlled. The punch velocities are indicated with description boxes inside the graph for the three bundles of DC simulations.

The three experiments with the lowest punch velocity of 0.04 mm∙s^−1^ are also used to analyze the Young’s modulus with the response of the unloading state and show good agreement between experiments and simulations. The strain values and strain rates are investigated for the two loading types, as well as all temperatures and punch velocities. The results are summarized in Figure 8.

The force-controlled simulations reveal versatile strain rates throughout each individual test from 2 × 10^−3^ to 7 × 10^−6^ s^−1^, whereas the displacement-controlled simulations show almost constant strain rates ranging from 3 × 10^−4^ to 5 × 10^−2^ s^−1^ for the different punch velocities. The distribution of maximum principal strain is depicted in Figure 8a for the temperature 900 °C, punch velocity 0.04 mm∙s^−1^, and two representative displacement states. The present strain rates are calculated at the evaluation point “strain” indicated with a yellow cross, which represents the point of highest strain throughout the specimen. Therefore, the strain rates in Figure 8b represent an upper bound for the whole specimen. The material model is parameterized to give an accurate response to each of the loading types, but the agreements of the force-controlled experiments and the displacement-controlled experiments with the lowest punch velocity of 0.04 mm∙s^−1^ are weighted higher. The fact that the gradients in strain and strain rates change for the different conditions ensures that the strain rate sensitivity is uniquely determinable. The resulting material model is capable of reproducing the material behavior for the whole range of strain rates present at the straightening stage of the continuous casting process. The modelled material behavior outside the validated definition range of strain rates between 5 × 10^−2^ and 7 × 10^−6^ s^−1^ is not relevant for the process and is therefore not investigated in detail.

Table 2 summarizes the material model parameters for the three representative temperature states. The notations are adopted from [12] and the governing equations are summarized in Section 2.2.2, see Equations (1)–(6). To ensure the usability of the complete set of parameters over temperature in non-isothermal calculations, an eye was set on the individual parameter’s monotonic evolution with temperature. This is only broken for the three parameters, m1, m3, and n around 900 °C, which indicates slightly different underlying mechanisms governing the time-dependent material behavior in this temperature regime. The initial yield stress for all three temperatures investigated is zero. This fact allows viscoplastic effects to occur even at low von Mises stress states, which was observed in the experiments.

Although the presented data is determined for a 0.17 wt.% C steel, it is expected that the data also provides a good approximation for other low-carbon steel grades. However, the chemical composition and the processing conditions will have an effect on viscoplastic material behavior. Understanding these interactions and its influence on the material data will be the focus of future investigations.

## 4. Conclusions 

An elasto-viscoplastic material model was parameterized for a 0.17% C steel with a close relation to the continuous casting process. The main findings and modelling achievements can be summed up as follows:

- The microstructure and mechanical behavior greatly differ between directly-cast and reheated samples. The time-dependent mechanical responses vary by a factor of up to about two between these material states.
- The present work for the first time provides a material model for a directly-cast microstructure which is very similar to the material state in a continuous casting strand shell.
- The viscoplastic model accounts for cyclic plasticity effects. Considering cyclic material effects are crucial for accurate modelling, e.g., the straightening stage in continuous casting where numerous rolls introduce cyclic loading to the strand shell.

The parameterized model allows for realistic mechanical simulation analyses of the straightening stage in a continuous casting process, such as investigations of local strain rates, local strain, and local stress, and it enables the evaluation of crack initiation limits and crack driving forces of surface cracks.

## Figures and Tables

**Table 1:** Composition of the investigated steel grade (wt.%).

C	Si	Mn	P	S	Al	Fe
0.17	0.4	1.55	0.01	<0.004	0.03	balance

**Table 2:** Material model parameters for the three representative temperatures 800, 900, and 1000 °C.

	Temperature [°C]	800	900	1000
Elastic	Young’s modulus [MPa]	25,000	18,000	12,500
	Poisson [-]	0.34	0.35	0.36
Initial yield stress	R0 [MPa]	0	0	0
Kinematic hardening	C1 [MPa]	7500	6000	2500
	γ1 [-]	600	600	600
	C2 [MPa]	3500	2300	1500
	γ2 [-]	180	180	180
	C3 [MPa]	400	300	200
	γ3 [-]	30	30	30
Isotropic hardening	Q [MPa]	80	50	50
	b [-]	6.5	6	1
Static recovery	M1 [MPa]	500	300	100
	m1 [-]	3.8	2.6	3
	M2 [MPa]	320	240	180
	m2 [-]	2.8	2.5	1.8
	M3 [MPa]	400	250	250
	m3 [-]	3.45	3.3	3.5
Viscoplastic potential	K [MPa]	40	56	75
	n [-]	3.2	2.8	3.4
